# Association of Non-alcoholic Fatty Liver Disease With Salt Intake and Dietary Diversity in Chinese Medical Examination Adults Aged 18–59 Years: A Cross-Sectional Study

**DOI:** 10.3389/fnut.2022.930316

**Published:** 2022-07-12

**Authors:** Xiaofei Luo, Ying Li, Yi Zhou, Chun Zhang, Lijun Li, Yating Luo, Jiangang Wang, Yinglong Duan, Jianfei Xie

**Affiliations:** ^1^Health Management Center, The Third Xiangya Hospital of Central South University, Changsha, China; ^2^Xiangya Nursing School, Central South University, Changsha, China; ^3^Department of Nursing, The Third Xiangya Hospital of Central South University, Changsha, China

**Keywords:** non-alcoholic fatty liver disease, salt intake, dietary diversity, adults, medical examinations

## Abstract

**Objectives:**

Given the significance of dietary factors in the development of non-alcoholic fatty liver disease (NAFLD). We conducted a cross-sectional study to investigate the association of NAFLD with salt intake and dietary diversity in a medical examination population aged 18–59 years.

**Methods:**

Data from two Chinese health management centers were utilized between January 2017 and December 2019. The general information, laboratory tests, lifestyle habits, and diet of the participants were all evaluated. Based on alcohol consumption and abdominal ultrasound results, a total of 23,867 participants were divided into the NAFLD (*n* = 7,753) and control (*n* = 16,114) groups. Salt intake and dietary diversity were calculated separately for study participants using the spot urine method and dietary diversity scores (DDS). The multilevel logistic model and subgroup analysis were used to analyze the relationship between salt intake, dietary diversity, and NAFLD.

**Results:**

We found that the prevalence of NAFLD was 32.48%. Salt intake was associated with increased NAFLD (Q2 vs. Q1: OR = 1.201, 95% CI 1.094-1.317, *P* < 0.001; Q3 vs. Q1: OR = 1.442, 95% CI 1.316-1.580, *P* < 0.001; Q4 vs. Q1: OR = 1.604, 95% CI 1.465-1.757, *P* < 0.001), whereas sufficient dietary diversity was a protective factor for NAFLD (Sufficient DDS vs. Insufficient DDS: OR: 0.706, 95% CI 0.517-0.965, *P* < 0.05). The effects of salt intake and dietary diversity on NAFLD were equally stable in the subgroup analysis.

**Conclusions:**

We can conclude that NAFLD is highly prevalent in medical examination adults aged 18-59 years in China. Furthermore, the risk of salt intake for NAFLD and the protective effect of dietary diversity on NAFLD should be taken into account in the management of NAFLD.

## Introduction

Non-alcoholic fatty liver disease (NAFLD) is a metabolic syndrome characterized by abnormal accumulation of fatty material in the liver (1). NAFLD has become the leading cause of chronic liver disease worldwide, accounting for ~25% of the prevalence (2). More than 30% of the population in the United States is affected by NAFLD (3). The growth rate of NAFLD in China is expected to reach its highest point in 2030 (4). NAFLD is associated with adverse outcomes of liver diseases and chronic conditions such as cardiovascular disease and diabetes, making it significant global health and economic burden (5). The pathogenesis of NAFLD is complex, and metabolic disorder syndromes such as obesity, hypertension, and dyslipidemia are the main causative agents of NAFLD (3). Increasing evidence also suggests that dietary intake plays a vital role in developing NAFLD (6, 7).

High salt intake is a major dietary risk factor, resulting in ~1.8 million deaths worldwide in 2019 (8). A large-scale study of the global impact of diet on health noted that the daily salt intake of the Chinese population exceeds that of other countries and regions (9). Evidence has shown that reducing salt intake can prevent and control obesity and chronic conditions such as hypertension and cardiovascular disease (10). As a result, it is essential to focus on salt intake in the Chinese chronic disease population and analyze its association. Current studies on salt intake are usually conducted by self-perception reports and 24-h urine sodium measurements. Self-perception is less reliable, while 24-h urine sodium measurement is practically difficult to perform and is more suitable for application in small sample sizes (11). Casual spot urine estimation of 24-h urine sodium is the more commonly used method for estimating the level of salt intake in the population (12). Sodium is an essential nutrient for the body, but in asymptomatic adults, higher dietary sodium intake as measured by 24-h urine sodium can increase the development of NAFLD by contributing to obesity (13). Shen et al. (14) found that perceived high salt intake was also associated with a higher risk of NAFLD. However, no studies have directly explored casual spot urine estimates of salt intake and NAFLD in the Chinese population aged 18–59 years.

In addition, since most nutrients may interact with each other in the diet, it is not enough to consider the association of a single nutrient or food with NAFLD (15). Several previous studies have examined various dietary patterns and the risk of NAFLD (16–19). However, little has been reported on the relationship between dietary diversity and NAFLD. Dietary diversity is an essential indicator for evaluating the dietary quality index (20). Being based on food groups is more helpful in predicting nutritional adequacy than individual food indicators, although food groups vary considerably across studies (21). It was proposed that dietary diversity was positively correlated with micronutrient adequacy (22). Besides, a study conducted in Iran showed that higher dietary diversity was associated with higher quality dietary intake (23). It may be thought that subjects with higher quality diets may follow a healthier diet and therefore may be negatively associated with NAFLD. Currently, there is limited evidence, small sample sizes, and inconsistent findings regarding the relationship between dietary diversity and NAFLD, and no studies on dietary diversity and the risk of NAFLD in Chinese populations have been identified.

Salt intake and dietary diversity, as single nutrient and overall dietary quality evaluation indicators, respectively, may be combined to better evaluate the role of dietary factors on NAFLD. Therefore, this study intends to investigate the relationship between salt intake, dietary diversity, and NAFLD in Chinese adults aged 18–59 years by analyzing data from medical examination centers and providing reference evidence for further development of dietary management guidelines for NAFLD.

## Materials and Methods

### Study Design and Participants

**Figure 1:**
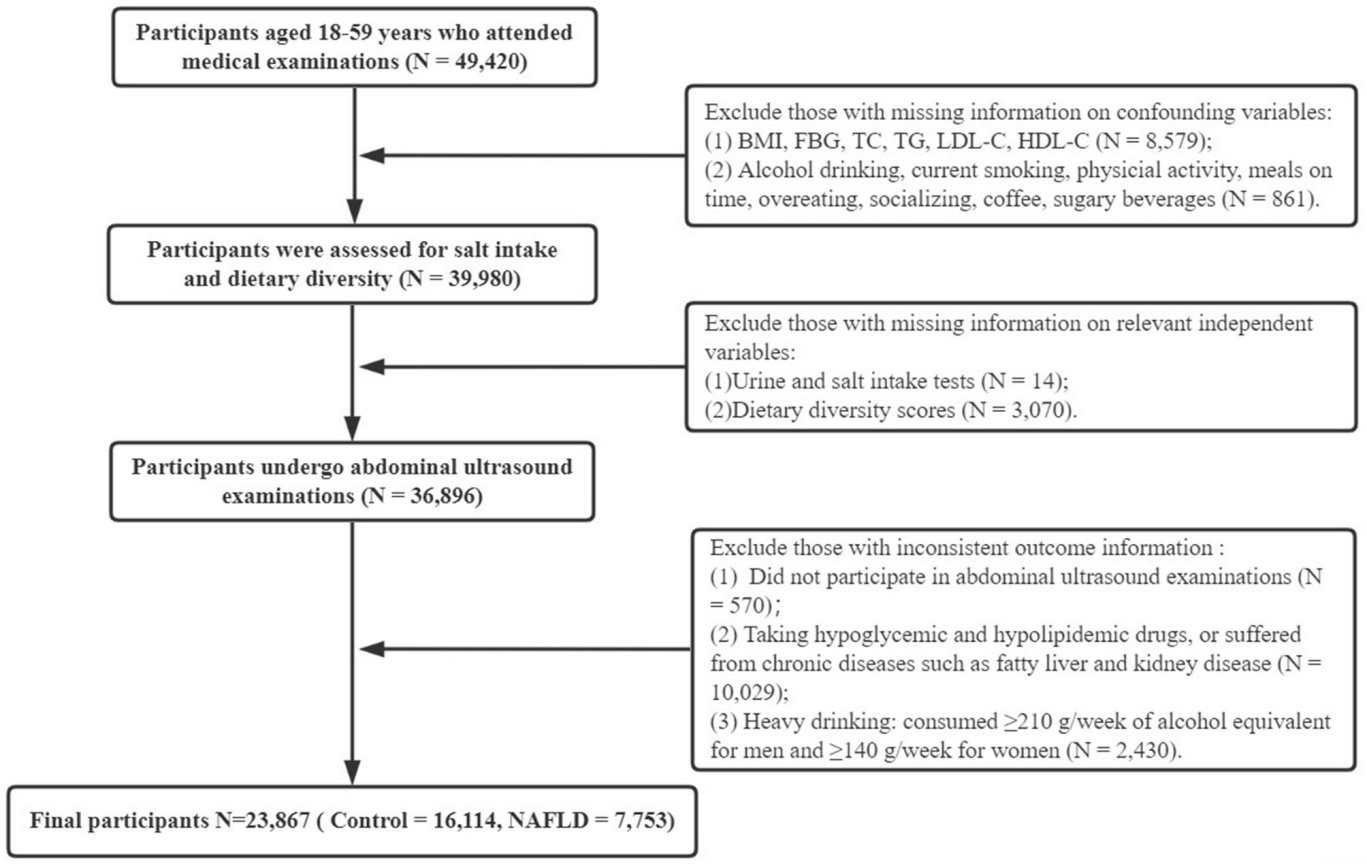
Flowchart of the study. BMI, body mass index; FBG, fasting blood glucose; TC, total cholesterol; TG, triglycerides; LDL-C, low-density lipoprotein cholesterol; HDL-C, high-density lipoprotein cholesterol; NAFLD, non-alcoholic fatty liver disease.

Data for this cross-sectional survey were obtained from two general tertiary care medical examination centers in China. A total of 49,420 adults underwent health examinations from 1 January 2017 to 31 December 2019. Inclusion criteria were (1) age between 18 and 59 years and (2) completion of all general information, clinical and laboratory tests, lifestyle habits and dietary surveys, and abdominal ultrasonography. Exclusion criteria included (1) use of hypoglycemic and hypolipidemic drugs; (2) significant abnormal liver function and other diseases affecting liver fat content, hematologic diseases, kidney disease, and those with existing related chronic diseases; and (3) history of excessive alcohol consumption (alcohol equivalent ethanol consumption ≥210 g/week for men and ≥140 g/week for women) (24). All included participants were divided into the NAFLD group and the control group by abdominal ultrasound results. After data cleaning, 23,867 participants were finally enrolled, and the detailed participant selection process is shown in Figure 1. Ethical approval for the study was obtained from the relevant hospitals (No. 2020-S587), and all participants voluntarily signed a written informed consent form.

### Data Collection and Measurements

#### General Characteristics, Clinical and Laboratory Tests, and Lifestyle Habits

General characteristics were collected, including age and sex. Age was divided into the youth group (18–44 years) and the middle-aged group (45–59 years) according to the World Health Organization's classification of the population.

Clinical and laboratory tests were examined by uniformly trained professionals using nationally certified instruments after an overnight fast, including height and weight and blood tests. Body mass index (BMI) was calculated as follows: weight (kg)/height^2^ (m^2^). BMI was classified according to the Chinese health industry standards into lean, BMI < 18.5 kg/m^2^; normal, BMI between 18.5–23.9 kg/m^2^; overweight, 24.0–27.9 kg/m^2^; and obese, BMI ≥ 28 kg/m^2^ (25). Blood indicators measured participants' fasting blood glucose (FBG), total cholesterol (TC), triacylglycerol (TG), low-density lipoprotein cholesterol (LDL-C), and high-density lipoprotein cholesterol (HDL-C). Hyperglycemia was defined as FBG ≥ 7.0 mmol/L (26). Dyslipidemia was considered as meeting one of the conditions, TC ≥ 5.2 mmol/L, TG ≥ 1.7 mmol/L, LDL-C ≥ 3.4 mmol/L, or HDL-C < 1.0 mmol/L (27).

The lifestyle habits were collected through self-report by participants. The questionnaire consisted of four components: alcohol drinking, current smoking, physical activity, and dietary habits. Alcohol drinking was defined as those who consumed <210 g/week of alcohol equivalent for men and <140 g/week for women (24). Never smoked and quit smoking were classified as non-smoking. Physical activity assessed whether individuals exercised in their leisure time and was defined as “yes” ≥3 times/week for ≥30 mins each time (28). In addition, five common related risky diet habits were investigated: meals on time, overeating, socializing, coffee, and sugary beverages, with a “yes” or “no” response for each section.

#### Assessment of Salt Intake

The salt intake of participants was estimated by the spot urine sodium method. It is convenient, operational, and currently recognized as one of the reliable indicators of salt intake (11). Spot urine samples were collected from the participants, and urine creatinine, sodium, and potassium concentrations were measured using the ion electrode method. Then, the 24-h urine sodium excretion was estimated using Tanaka's (29) formula to infer the salt intake.

#### Dietary Diversity Scores

The dietary diversity was captured by the dietary diversity score (DDS), which has been well-validated and widely used in the Chinese population (30, 31). The DDS was developed according to the WHO and the Chinese Dietary Guidelines and the Balanced Diet Pagoda and assessed the consumption of nine food groups: grains, vegetables, fruits, livestock meat, fish and shrimp, eggs, milk and dairy products, beans, and oils and fats (32, 33). The calculation of DDS was based on participants' recall of the number of food groups consumed in the past three days, with one point for each food group consumed, without counting the frequency or quantity of intake. The scores ranged from 0 to 9 and could be divided into three levels (30): insufficient (score of 0–3), moderate (score of 4–6), and sufficient (score of 7–9).

#### Determination of NAFLD

The ultrasound diagnostic criteria for NAFLD were referenced from the Chinese Guidelines for the Treatment of NAFLD (2018 Updated Version) (24). First, participants who already had abnormal liver function or disease affecting liver fat content were excluded, as well as those who consumed alcohol ≥210 g/week for men and ≥140 g/week for women. Next, qualified physicians performed fasting abdominal ultrasound examinations on the examinee and issued a report. The findings of the report were signed by the responsible physician and entered into the system. Depending on the reported results, participants were divided into two groups: NAFLD patients and controls.

### Statistical Analysis

Statistics and analysis were conducted using SPSS version 25.0. Exploratory analysis was used to test normality for continuous variables. Descriptive statistics were expressed as medians [interquartile range (IQR)] or frequencies (percentages). The chi-square tests and Mann–Whitney tests were used to determine whether the participants' characteristics, clinical laboratory tests, and diet differed among patients with NAFLD. The direct correlation between salt intake, dietary diversity, and NAFLD was tested by the Mann–Whitney test, chi-square test, and Kruskal–Wallis test. Multilevel binary logistic regression analysis assessed the relationship between salt intake, dietary diversity, and NAFLD by constructing four models controlling sequentially for confounding variables. The crude model was the unadjusted model; Model 1 added age and sex; Model 2 added clinical and laboratory results on BMI, hyperglycemia, and dyslipidemia to Model 1; and Model 3 was a complete model with lifestyle habits including alcohol drinking, current smoking, physical activity, meals on time, overeating, socializing, coffee, and sugary beverages added to Model 2. Subgroup analysis was then performed by age, sex, BMI, hyperglycemia, dyslipidemia, alcohol drinking, current smoking, physical exercise, socializing, sugary beverages, and salt intake/DDS to further explore the relationship between salt intake, dietary diversity, and NAFLD. The odds ratio (OR) and 95% confidence interval (95% CI) were calculated, and *P* < 0.05 was considered significant for all analyses.

## Results

### Participants' Characteristics and the Prevalence of NAFLD

Of the 23,867 adults aged 18–59 years included in this study, 7,753 participants had NAFLD, with an incidence of 32.48% (see Table 1). The majority of participants were assessed at the medical examination center 1 (91.4%). The age of participants had a median and IQR of [43 (35, 50)]; 56.0% of participants were aged 18–44 years; 45.6% were female. Overweight or obese individuals made up 47.1% of participants, and more than half of participants had abnormal lipids (56.4%), while a mere 3.4% were hyperglycemia. Concerning lifestyle, most participants did not drink alcohol (72.9%), did not smoke (77.7%), did not overeat (93.6%), and did not socialize (80.3%). Physical exercise, meals on time, and non-sugary beverages accounted for the majority of participants, at 66.5%, 65.5%, and 51.7%, respectively. Only 27.5% of the participants drank coffee.

**Table 1:** Basic characteristics of study participants stratified by NAFLD.

**Variables**	**Total (*N* = 23,867)**	**Control (*N* = 16,114, 67.52%)**	**NAFLD (*N* = 7,753, 32.48%)**	***P-*value**
**Level 1 General characteristics**				
Examination center				0.502
Center 1	21,818 (91.4)	14,717 (67.5)	1,397 (68.2)	
Center 2	2,049 (8.6)	7,101 (32.5)	652 (31.8)	
Age (years)	43 (35, 50)	42 (33, 49)	45 (37, 51)	<0.001^***^
Age by category				<0.001^***^
18–44	13,371 (56.0)	9,603 (59.6)	3,768 (48.6)	
45–59	10,496 (44.0)	6,511 (40.4)	3,985 (51.4)	
Sex				<0.001^***^
Male	12,988 (54.4)	7,370 (45.7)	5,618 (72.5)	
Female	10,879 (45.6)	8,744 (54.3)	2,135 (27.5)	
**Level 2 Clinical and laboratory tests**				
BMI (kg/m^2^)	23.8 (21.6, 26.0)	22.8 (20.9, 24.7)	25.9 (24.0, 27.8)	<0.001^***^
BMI by category^a^				<0.001^***^
Lean	738 (3.1)	696 (4.3)	42 (0.5)	
Normal	11,899 (49.9)	10,051 (62.4)	1,848 (23.8)	
Overweight	8,658 (36.3)	4,538 (28.2)	4,120 (53.1)	
Obese	2,572 (10.8)	829 (5.1)	1,743 (22.5)	
FBG (mmol/L)	5.3 (4.9, 5.6)	5.2 (4.9, 5.5)	5.4 (5.1, 5.9)	<0.001^***^
Hyperglycemia^b^				<0.001^***^
None	23,057 (96.6)	15,815 (98.1)	7,242 (93.4)	
Yes	810 (3.4)	299 (1.9)	511 (6.6)	
TC (mmol/L)	4.9 (4.4, 5.6)	4.8 (4.3, 5.4)	5.2 (4.6, 5.8)	<0.001^***^
TG (mmol/L)	1.3 (0.9, 2.0)	1.1 (0.8, 1.6)	2.0 (1.4, 2.9)	<0.001^***^
HDL-C (mmol/L)	1.3 (1.1, 1.5)	1.4 (1.2, 1.6)	1.2 (1.0, 1.3)	<0.001^***^
LDL-C (mmol/L)	2.8 (2.3, 3.3)	2.8 (2.3, 3.3)	2.9 (2.4, 3.4)	<0.001^***^
Dyslipidemia^c^				<0.001^***^
None	10,402 (43.6)	8,723 (54.1)	1,679 (21.7)	
Yes	13,465 (56.4)	7,391 (45.9)	6,074 (78.3)	
**Level 3 Lifestyle habits**				
Alcohol drinking				<0.001^***^
None	17,389 (72.9)	12,527 (77.7)	4,862 (62.7)	
Yes^d^	6,478 (27.1)	3,587 (22.3)	2,891 (37.3)	
Current smoking				<0.001^***^
None	18,546 (77.7)	13,250 (82.2)	5,296 (68.3)	
Yes	5,321 (22.3)	2,864 (17.8)	2,457 (31.7)	
Physical activity				<0.001^***^
None	8,003 (33.5)	5,231 (32.5)	2,772 (35.8)	
Yes	15,864 (66.5)	10,883 (67.5)	4,981 (64.2)	
Meals on time				0.043^*^
None	8,237 (34.5)	5,631 (34.9)	2,606 (33.6)	
Yes	15,630 (65.5)	10,483 (65.1)	5,147 (66.4)	
Overeating				<0.001^***^
None	22,339 (93.6)	15,197 (94.3)	7,142 (92.1)	
Yes	1,528 (6.4)	917 (5.7)	611 (7.9)	
Socializing				<0.001^***^
None	19,168 (80.3)	13,392 (83.1)	5,776 (74.5)	
Yes	4,699 (19.7)	2,722 (16.9)	1,977 (25.5)	
Coffee				0.011^*^
None	17,304 (72.5)	11,765 (73.0)	5,539 (71.4)	
Yes	6,563 (27.5)	4,349 (27.0)	2,214 (28.6)	
Sugary beverages				<0.001^***^
None	12,333 (51.7)	8,560 (53.1)	3,773 (48.7)	
Yes	11,534 (48.3)	7,554 (46.9)	3,980 (51.3)	

*Continuous variables were shown as numbers (percentages); Categories variables were expressed as median [interquartile range (IQR)]); Percentage sums of categorical variables may not equal 100% due to rounding; P-Values were derived from Mann–Whitney tests for continuous variables and chi-square tests for categorical variables. NAFLD, non-alcoholic fatty liver disease; BMI, body mass index; FBG, fasting blood glucose; TC, total cholesterol; TG, triglyceride; LDL-C, low-density lipoprotein cholesterol; HDL-C, high-density lipoprotein cholesterol*.

a *Lean means BMI < 18.5 kg/m^2^, normal means BMI between 18.5–23.9 kg/m^2^, overweight means BMI between 24.0–27.9 kg/m^2^, and obese means BMI ≥ 28.0 kg/m^2^*;

b *Hyperglycemia was defined as blood glucose ≥ 7.0 mmol/L*.

c *Dyslipidemia was defined as meeting one of these criteria: TC ≥ 5.2 mmol/L, TG ≥ 1.7 mmol/L, LDL-C ≥ 3.4 mmol/L, and HDL-C < 1.0 mmol/L*;

d *The answer “Yes” to alcohol drinking represents alcohol consumption equivalent to <210 g/week of ethanol for men and <140 g/week for women*;

* *P < 0.05*;

*** *P < 0.001*.

### Univariate Analysis of NAFLD

The results of the univariate analysis of NAFLD are also shown in Table 1. There was no statistically significant difference between participants from different medical examination centers and having NAFLD (*P* = 0.502). In terms of general characteristics, participants with NAFLD were more likely to be men, older, and 45–59 years old (middle age) compared to those without NAFLD (*P* < 0.001). The clinical and laboratory results indicated that participants who were overweight or obese, hyperglycemia, and dyslipidemia were at higher risk for NAFLD (*P* < 0.001). Participants who did not have NAFLD had lower TC [4.8 (4.3, 5.4) mmol/L], TG [1.1 (0.8, 1.6) mmol/L], LDL-C [2.8 (2.3, 3.3) mmol/L], and higher HDL-C [1.4 (1.2, 1.6) mmol/L]. Among lifestyles, participants with NAFLD reported much higher alcohol drinking, current smoking, overeating, socializing, sugary beverages, and less physical activity (*P* < 0.001). In addition, the association of meals on time and coffee with the finding of NAFLD was also statistically significant (*P* < 0.05).

### Distribution and Association Between Salt Intake, Dietary Diversity, and NAFLD

As shown in Table 2, the median and IQR of the urine creatinine, urine sodium, urine potassium, and urine sodium-potassium ratios were [11,288 (4,767, 14,594)], [116.3 (68.3, 158.6)], [48.7 (27.5, 71.1)], and [2.3 (1.6, 3.3)], respectively. The analysis results showed that participants with NAFLD had significantly higher urine creatinine, sodium, potassium, sodium-potassium ratio, and salt intake than those without NAFLD (*P* < 0.001).

**Table 2:** Distribution of salt intake and dietary diversity among participants with and without NAFLD.

**Variables**	**Total (*N* =23,867)**	**Control (*N* = 16,114, 67.52%)**	**NAFLD (*N* = 7,753, 32.48%)**	***P*-value**
Urine creatinine	11,288 (4,767, 14,594)	9,849 (4,127, 14,183)	13,136 (6,974, 15,621)	<0.001^***^
Urine sodium	116.3 (68.3, 158.6)	107.2 (59.5, 151.9)	133.0 (90.0, 169.3)	<0.001^***^
Urine potassium	48.7 (27.5, 71.1)	45.1 (24.9, 69.1)	54.7 (34.6, 74.9)	<0.001^***^
Sodium-potassium ratio	2.3 (1.6, 3.3)	2.3 (1.6, 3.3)	2.4 (1.7, 3.5)	<0.001^***^
Salt intake	8.3 (7.1, 9.5)	8.1 (6.9, 9.3)	8.7 (7.5, 9.8)	<0.001^***^
Grains	22.568 (94.6)	15,299 (94.9)	7,269 (93.8)	<0.001^***^
Vegetables	18,576 (77.8)	12,566 (78.0)	6,010 (77.5)	0.419
Fruits	23,116 (96.9)	15,663 (97.2)	7,453 (96.1)	<0.001^***^
Livestock meat	18,059 (75.7)	11,947 (74.1)	6,112 (78.8)	<0.001^***^
Fish and shrimp	22,572 (94.6)	15,250 (94.6)	7,322 (94.4)	0.529
Eggs	22,809 (95.6)	15,394 (95.5)	7,415 (95.6)	0.703
Milk and dairy products	15,383 (64.5)	10,625 (65.9)	4,758 (61.4)	<0.001^***^
Beans	10,768 (45.1)	7,376 (45.8)	3,392 (43.8)	0.003^**^
Oils and fats	20,300 (85.1)	13,519 (83.9)	6,781 (87.5)	<0.001^***^
DDS^a^				0.022^*^
Insufficient	230 (1.0)	136 (0.8)	94 (1.2)	
Moderate	5,414 (22.7)	3,674 (22.8)	1,740 (22.4)	
Sufficient	18,223 (76.4)	12,304 (76.4)	5,919 (76.3)	

*Continuous variables were shown as numbers (percentages); Categories variables were expressed as median [interquartile range (IQR)]; Percentage sums of categorical variables may not equal 100% due to rounding; The unit of urine creatinine is umol/L, the unit of urine sodium and potassium is mmol/L, and the unit of salt intake is g/d.; P-Values were derived from Mann–Whitney tests for continuous variables and chi-square tests for categorical variables. DDS, dietary diversity scores; NAFLD, non-alcoholic fatty liver disease*.

a *Insufficient refers to a score of 0–3, Moderate refers to a score of 4–6 and Sufficient refers to a score of 7–9*;

* *P < 0.05*;

** *P < 0.01*;

*** *P < 0.001*.

The food group most consumed among participants was fruit (96.9%), followed by eggs (95.6%), fish and shrimp (94.6%), and grains (94.6%), with the least consumed being beans (45.1%). Participants with NAFLD consumed fewer grains (93.8%), fruits (96.1%), milk and dairy products (61.4%), and beans (43.8%) and consumed more livestock meat (78.8%), oils and fats (87.5%). This suggests that the former may reduce the risk of NAFLD, while the latter may be considered a risk factor for NAFLD (*P* < 0.01).

**Figure 2:**
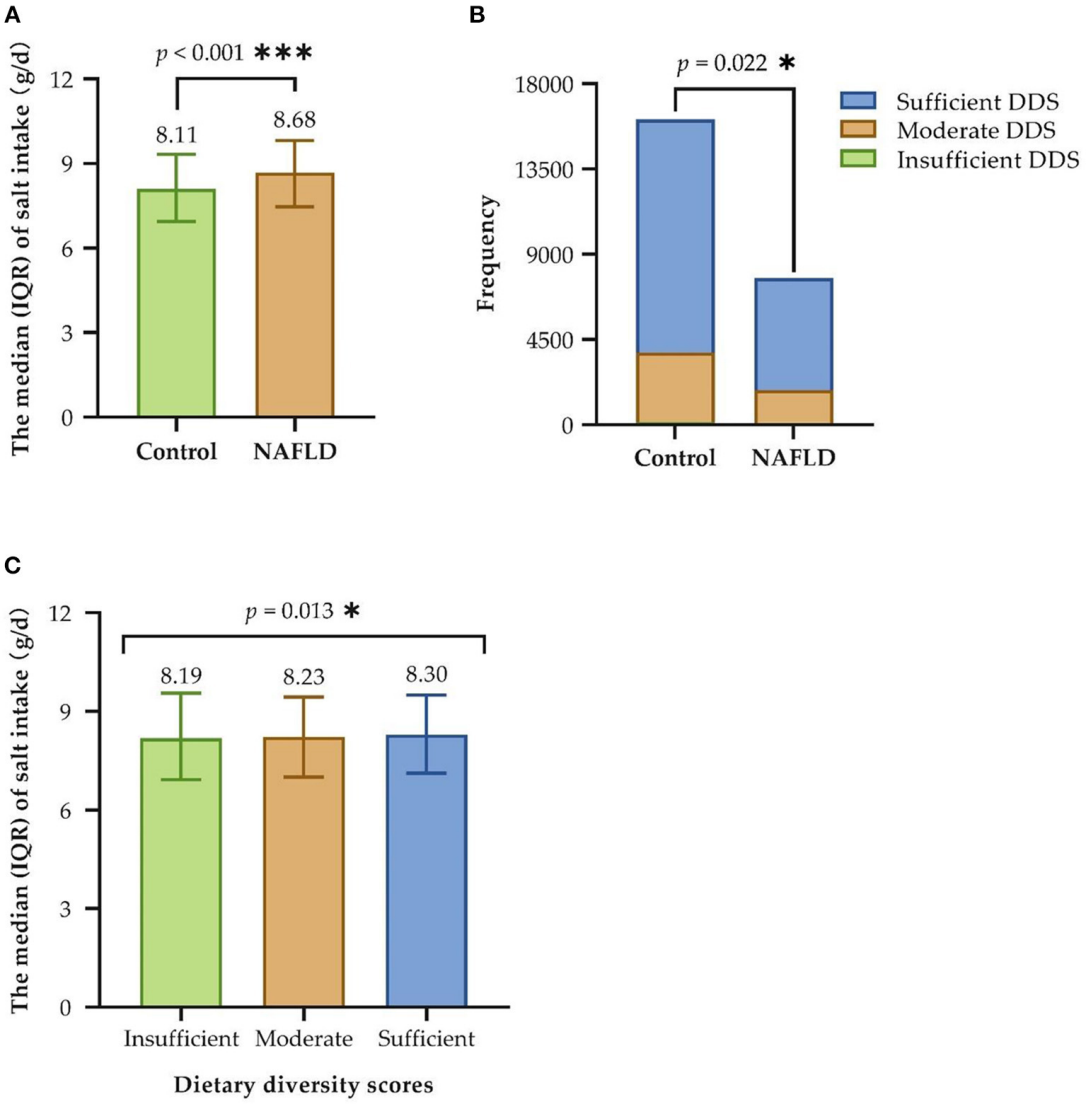
Correlation between salt intake, dietary diversity, and NAFLD. **(A)** Univariate analysis of salt intake and NAFLD; **(B)** Univariate analysis of dietary diversity and NAFLD; **(C)** Univariate analysis of salt intake and dietary diversity. IQR, interquartile range; DDS, dietary diversity scores; NAFLD, non-alcoholic fatty liver disease; Insufficient refers to a score of 0–3, Moderate refers to a score of 4–6 and Sufficient refers to a score of 7–9; *P < 0.05; ***P < 0.001.

Regarding the main variables (see Table 2; Figure 2), the median and IQR of salt intake was [8.3 (7.1, 9.5)], and dietary diversity was achieved by 76.4% of the participants. The correlation between high salt intake in patients with NAFLD was significant (*P* < 0.001). A greater proportion of patients with NAFLD had an insufficient DDS, and fewer had a moderate and sufficient DDS than the control group (*P* = 0.022). Furthermore, the median salt intake in the insufficient, moderate, and sufficient groups was 8.19, 8.23, and 8.30, respectively. The Kruskal–Wallis test indicated a significant direct correlation between dietary diversity and salt intake (*P* = 0.013).

### Multilevel Logistic Regression of NAFLD With Salt Intake and Dietary Diversity

The multilevel binary logistic regression model was constructed with salt intake and dietary diversity as independent variables and NAFLD as the outcome variable (see Table 3). Both the unadjusted and all adjusted models indicated that salt intake was a risk factor for NAFLD, and the likelihood of developing NAFLD increased with high quartiles of salt intake (*P* < 0.001). Compared to the lowest quartile group, the odds ratio for NAFLD decreased with higher salt intake after adjusting for confounders. The odds ratio [95% confidence interval] for the fourth quartile of salt intake in the crude model and the full Model 3 was 2.071 [1.915–2.240] and 1.604 [1.465–1.757], respectively.

**Table 3:** Multilevel logistic regression of NAFLD with salt intake and dietary diversity.

**Variables**	**Odds ratio [95% confidence interval]**			
	**Crude**	**Model 1**	**Model 2**	**Model 3**
Level 0 Main independent variables				
Salt intake				
Quartile 1	Ref	Ref	Ref	Ref
Quartile 2	1.294^***^ [1.194–1.403]	1.253^***^ [1.152–1.362]	1.181^***^ [1.077–1.295]	1.201^***^ [1.094–1.317]
Quartile 3	1.689^***^ [1.560–1.828]	1.638^***^ [1.509–1.779]	1.416^***^ [1.293–1.550]	1.442^***^ [1.316–1.580]
Quartile 4	2.071^***^ [1.915–2.240]	1.999^***^ [1.843–2.169]	1.582^***^ [1.446–1.731]	1.604^***^ [1.465–1.757]
DDS				
Insufficient	Ref	Ref	Ref	Ref
Moderate	0.681^**^ [0.519–0.893]	0.698^*^ [0.527–0.926]	0.734 [0.536–1.007]	0.733 [0.534–1.005]
Sufficient	0.683^**^ [0.523–0.893]	0.686^**^ [0.520–0.905]	0.705^*^ [0.517–0.962]	0.706^*^ [0.517–0.965]
Level 1 General characteristics				
Age (years)				
18–44		Ref	Ref	Ref
45–59		1.664^***^ [1.571–1.761]	1.377^***^ [1.292–1.467]	1.418^***^ [1.329–1.514]
Sex				
Male		Ref	Ref	Ref
Female		0.312^***^ [0.294–0.332]	0.575^***^ [0.537–0.616]	0.685^***^ [0.636–0.739]
Level 2 Clinical and laboratory tests				
Body mass index				
Thin			Ref	Ref
Normal			2.003^***^ [1.452–2.763]	2.034^***^ [1.474–2.807]
Overweight			7.322^***^ [5.306–10.103]	7.499^***^ [5.433–10.353]
Obese			15.199^***^ [10.919–21.156]	15.421^***^ [11.071–21.478]
Hyperglycemia				
None			Ref	Ref
Yes			2.545^***^ [2.158–3.002]	2.596^***^ [2.198–3.065]
Dyslipidemia				
None			ref	Ref
Yes			3.117^***^ [2.912–3.336]	3.000^***^ [2.802–3.213]
Level 3 Lifestyle habits				
Alcohol drinking				
None				Ref
Yes				1.335^***^ [1.240–1.438]
Current smoking				
None				Ref
Yes				1.239^***^ [1.146–1.341]
Physical activity				
None				Ref
Yes				0.831^***^ [0.777–0.889]
Meals on time				
None				Ref
Yes				1.053 [0.983–1.128]
Overeating				
None				Ref
Yes				0.993 [0.875–1.128]
Socializing				
None				Ref
Yes				1.102^*^ [1.016–1.195]
Coffee				
None				Ref
Yes				0.967 [0.899–1.040]
Sugary beverages				
None				Ref
Yes				1.154^***^ [1.080–1.234]

*Crude, unadjusted; Model 1, adjusted for age, sex; Model 2, Model 1 + BMI, hyperglycemia, dyslipidemia; Model 3: full model (i.e., Model 2 + alcohol drinking, current smoking, physical activity; meals on time, overeating, socializing, coffee, and sugary beverages). NAFLD, non-alcoholic fatty liver disease; DDS, dietary diversity scores*.

* *P < 0.05*;

** *P < 0.01*;

*** *P < 0.001*.

Additionally, the crude and adjusted models showed that dietary diversity was a protective factor for NAFLD (*P* < 0.05). An increase in DDS with more sufficient dietary diversity reduced the risk of NAFLD. Both moderate DDS and sufficient DDS versus insufficient DDS in the crude model (0.681 [0.519–0.893], *P* < 0.01; 0.683 [0.523–0.893], *P* < 0.01) and Model 1 (0.698 [0.527–0.926], *P* < 0.01; 0.686 [0.520–0.905], *P* < 0.01) after adjusting for age and sex showed significant odds ratios. Model 2 (0.705 [0.517–0.962], *P* < 0.05) and Model 3 (0.706 [0.517–0.965], *P* < 0.05) after continuing to adjust for BMI, hyperglycemia, dyslipidemia, alcohol drinking, current smoking, physical exercise, meals on time, overeating, socializing, coffee, and sugary beverages had a significant odds ratio to NAFLD for sufficient DDS over insufficient DDS.

### Subgroup Analysis of Salt Intake, Dietary Diversity, and NAFLD

**Figure 3:**
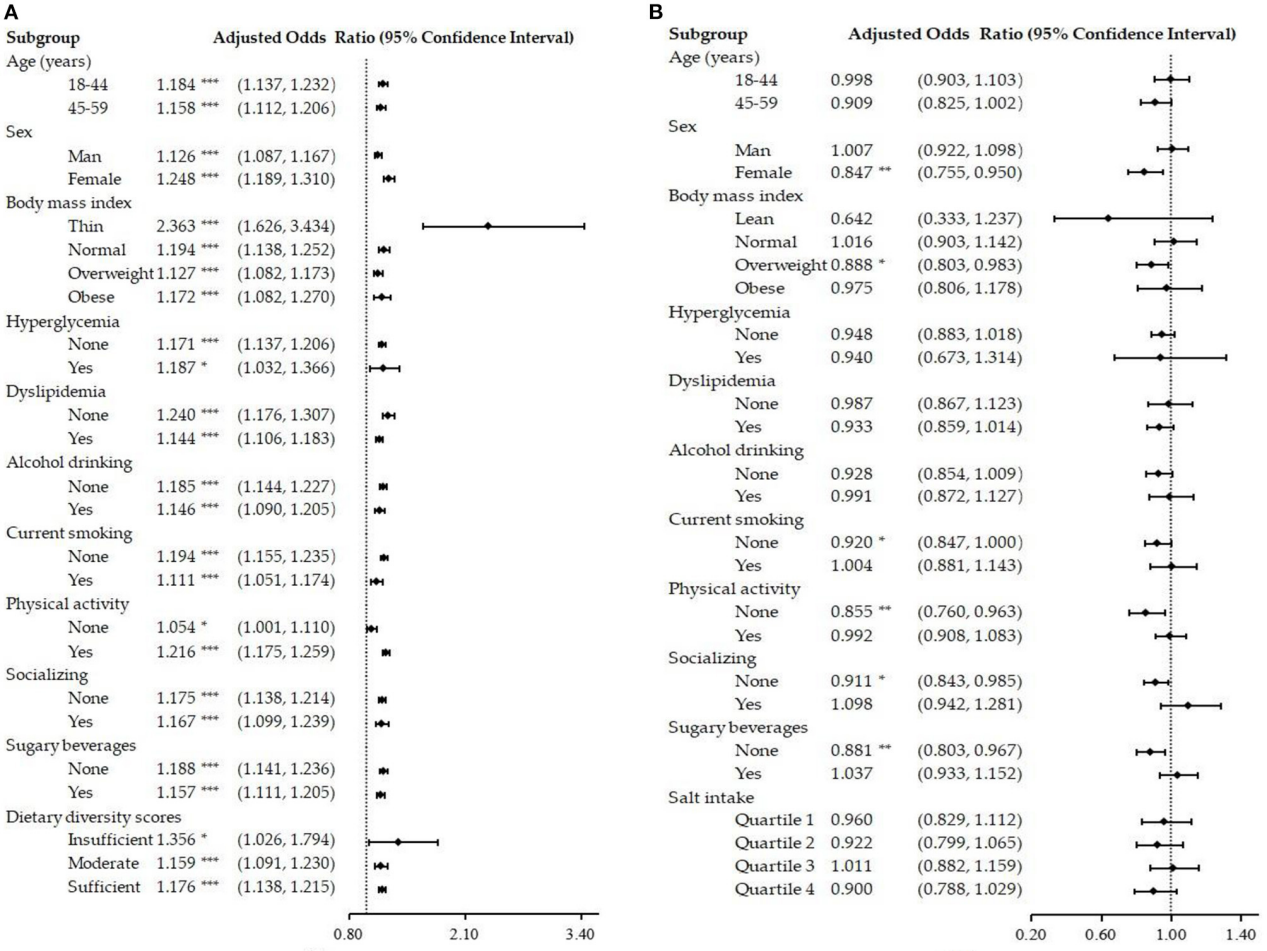
Subgroup analysis of the salt intake, dietary diversity, and NAFLD. **(A)** Association of salt intake with NAFLD after adjusting for other confounding variables; **(B)** Association of dietary diversity with NAFLD after adjusting for other confounding variables. NAFLD, non-alcoholic fatty liver disease; Insufficient refers to a score of 0–3, Moderate refers to a score of 4–6, and Sufficient refers to a score of 7–9; *P < 0.05; **P < 0.01; ***P < 0.001.

To further test the stability of the results, subgroup analyses were performed by age, sex, BMI, hyperglycemia, dyslipidemia, alcohol drinking, current smoking, physical exercise, socializing, and sugary beverages. An additional subgroup of DDS was added for salt intake and NAFLD, as well as a subgroup of salt intake for dietary diversity with NAFLD. Salt intake was presented as an independent risk factor for NAFLD in all subgroups after adjusted models (*P* < 0.05, see Figure 3A). Dietary diversity (see Figure 3B) was a protective factor for NAFLD in the female group, the overweight group, the current non-smoking group, the non-physical exercise group, the non-socializing group, and the non-sugary beverages group (*P* < 0.05).

## Discussion

This study was conducted to understand the relationship between salt intake, dietary diversity, and NAFLD in Chinese adult medical examination adults aged 18–59 years. The results showed that the prevalence of NAFLD among Chinese medical examination adults aged 18–59 years was 32.48%, which was significantly higher than that of a meta-analysis of 256,367 cases on the prevalence of NAFLD in China (20%) (34). In addition, we found that salt intake increased the risk of NAFLD, whereas dietary diversity was a protective factor for NAFLD, and the results were equally stable in the subgroup analysis.

NAFLD is typically a “lifestyle disease,” and the role of modifiable dietary factors in NAFLD is of increasing concern. The imbalance of nutrient intake can easily lead to overweight and obesity, increased blood glucose and lipid levels, and other health risks related to NAFLD (35). Studies have pointed out that dietary therapy can control the absorption of basal state free fatty acids, control postprandial hyperlipidemia, reduce insulin resistance, promote the metabolism and transport of lipoproteins, increase the number of antioxidants in the body, and adjust the dietary structure and balance (36). By preventing or eliminating the causes of fatty liver through reasonable and healthy dietary nutrition, the steatosis of early hepatocytes can be reversed while reducing the patient's symptoms (37). After the development of liver lesions in patients with NAFLD, even with tighter control of risk factors such as BMI, hyperglycemia, and dyslipidemia, only 17.8% of patients eventually reverse their liver lesions to normal (38). Therefore, early screening for dietary factors that can affect NAFLD and targeted prevention is essential to reduce the risk of developing NAFLD.

High salt intake is an essential dietary risk factor for disease burden in the population (39) and has a positive association with obesity, blood pressure, and cardiovascular disease (10, 40). Therefore, it may be associated with the development of NAFLD by the same pathway. Our study found the median and IQR of salt intake in adults aged 18–59 years to be (8.3 [7.1, 9.5]) g/d. This is well above the WHO and FAO recommended daily salt intake of <6 g/d (41). It is also higher than the Norwegian adult population study (8.05 g/d) (42). The results of logistic regression analysis further suggested that salt intake was a risk factor for NAFLD. After controlling for significant confounders in univariate analysis, the results still indicated that increased salt intake increased the risk of NAFLD. Although the mechanisms linking salt intake to NAFLD are unclear, researchers have suggested that insulin resistance may be a metabolic intermediate explaining the relationship between high salt intake and NAFLD (43). A study in rats showed that high salt intake enhances insulin sensitivity in adipocytes, improves glucose uptake and insulin-induced glucose metabolism, and promotes adipocyte hypertrophy (44). In addition, high salt intake activates the aldose reductase-fructokinase pathway in the liver and hypothalamus, leading to obesity, insulin resistance, and NAFLD (45). The significant role of obesity in the development of NAFLD may also be a potential mechanism for the association between salt intake and NAFLD. A high salt diet was found to be independently associated with elevated leptin levels in humans (46), and there was a direct positive correlation between salt intake and BMI or body fat percentage (13). Another study pointed out that high salt can also play a role in hepatic inflammation and fibrosis by modulating the renin-angiotensin system, leading to the progression of NAFLD (47). This provides the supporting basis for the present study. Several previous studies have also found an association between salt and NAFLD. However, the estimation of salt intake is often performed by dietary surveys or 24-h urine sodium assessment rather than the direct calculation of salt intake (11). Spot urine estimation of 24-h urine sodium excretion is considered a simple, rapid, and generalizable method for estimating the level of salt intake in a population (12). This study is the first to analyze the relationship between salt intake and NAFLD by examining spot urine in a large sample of the Chinese medical examination population.

In terms of dietary diversity, participants with NAFLD in this study consumed fewer grains, fruits, milk and dairy products, beans, and more livestock meat, and oils and fats. However, diets that increase the intake of vegetables and fruits, beans, fish and yogurt, and whole grains and reduce foods with meat, salt, and trans fats benefit the organism (48). The Mediterranean diet, characterized by this diet, was associated with lower levels of systemic inflammation and a reduced incidence of NAFLD (49). It is recommended to increase the consumption of grains, fruits, milk and dairy products, and beans and reduce the intake of fats and livestock meats. Several other dietary patterns have been associated with NAFLD. Healthy dietary patterns and Dietary Approaches to Stop Hypertension can play a key role in the prevention and control of NAFLD (6, 16). In a study of Chinese adults, the “Animal Food” dietary pattern of food consumption was associated with an increased risk of NAFLD, and the “Grains-Vegetables” dietary pattern was related to a reduced risk of NAFLD (17). However, it has also been found that a vegetable-rich diet was not associated with NAFLD (18), and the present study also did not find an association between vegetables and NAFLD. In centrally obese adolescents, a Western dietary pattern characterized by consumption of red meat, refined grains, processed meats, and high-sugar beverages may increase the risk of NAFLD (50). A Greek study reported that the fast food-type dietary pattern was independently associated with higher odds of NAFLD due to high levels of C-reactive protein and unsaturated fatty acids (19). A Lebanese study noted that the high fruit diet group was also a major potential risk factor for NAFLD, while the traditional diet consisting of vegetables and legumes was negatively associated with the odds of NAFLD (51). It can be learned that different dietary patterns have different effects on NAFLD, and the evidence focusing on the overall quality of the diet and NAFLD is still weak.

The present study also suggested that dietary diversity, as a protective factor for NAFLD, decreased the risk of NAFLD development. Sufficient DDS was associated with a reduced risk of NAFLD compared to insufficient DDS. Some trial evidence suggested that greater food diversity affected the composition of the gut flora, thereby further improving immune function and health status (52). Previous studies have found that increased dietary diversity reduces the incidence of diseases closely related to NAFLD such as overweight, metabolic syndrome, and cardiovascular disease (31, 53, 54). In addition, higher DDS was related to healthier eating habits and better metabolic profiles. Increased dietary diversity leads to more opportunities to choose foods that are negatively associated with NAFLD. Ebrahimi et al. (55) noted that dietary diversity was correlated with increased vitamin C, calcium, and fiber, all of which were negatively associated with liver disease. Furthermore, dietary diversity may increase the intake of various micronutrients, dietary fiber, and some healthful phytochemicals, which are positively associated with dietary balance (56, 57). A wide variety of foods from the same and different food groups can provide vitamins, minerals, and other micronutrients necessary for the health of the body, thereby improving dietary patterns and optimizing the structure of the diet. The more established “second strike” theory of NAFLD points to the importance of oxidative stress in the development of NAFLD (58). Dietary diversity may increase the adequate intake of antioxidants in the diet (59) and may be associated with NAFLD. Notably, another study found that DDS increase was not significantly associated with NAFLD (15). This difference in results may be due to the inclusion of food groups with different assessment methods, different populations assessed, and smaller samples for dietary diversity in their included studies. Furthermore, dietary diversity is not focused on increasing the number of nutrients but rather on promoting diversity and balance among nutrients. It should be clarified that the dietary diversity of an individual does not indicate complete nutritional health. Dietary diversity can ensure the intake of all types of nutrients, but if a particular nutrient is consumed too much or not enough, it is an unreasonable dietary structure, affecting nutritional health.

This study also showed that NAFLD was related to higher age, considering an association with increased metabolic decrease (38). Men were also prone to NAFLD because they were more socially active in their careers, and many had drinking and smoking habits (35). Consistent with previous studies, NAFLD was linked to overweight/obesity, hyperglycemia, and dyslipidemia, with higher BMI, glucose, and lipids in the NAFLD group than in the control group (35, 58). Alcohol drinking and current smoking were also associated with NAFLD. Evidence has shown that patients with NAFLD should avoid alcohol consumption (60), and passive smoking increases NAFLD risk by ~1.38-fold (61). Conversely, physical activity can improve the metabolic function of all bodies, which helps to control BMI and improve insulin resistance, therefore reducing the incidence of NAFLD (36). In addition, socializing and drinking sugary beverages can also affect NAFLD. Additional socializing can lead to excess nutrients being stored in the liver and the formation of a fatty liver. Sugary beverages can increase the metabolic burden so may be a key driver of NAFLD (62). Interestingly, previous studies suggested a protective effect of meals on time and coffee consumption on NAFLD (63). However, this study found no association between meals on time and coffee consumption and NAFLD in the adjusted regression model. This may be related to other factors, such as different subjects consuming coffee with different types, amounts, and food frequencies. Moreover, the present study found that salt intake was associated with dietary diversity. This has not been described in previous studies. Even without considering the amount and frequency of food intake, a greater variety of food intake can affect salt intake levels. Increased dietary diversity is accompanied by the intake of more categories of foods, which implies that people with sufficient dietary diversity have an increased probability of consuming salt-rich foods. This suggests that in addition to managing single nutrients such as sodium, it is equally important to consider whether the overall dietary intake is balanced for NAFLD (15).

To exclude the effect of these factors on NAFLD, we performed further subgroup analysis that illustrated the stability of the results of this study. The results indicated that salt intake remained a risk factor for NAFLD in all subgroups. This suggests that salt intake must be reduced in the management of patients with NAFLD. The protective effect of dietary diversity on NAFLD was more pronounced in the female, overweight, non-current smoking, non-physical activity, non-socializing, and non-sugary beverage groups. This may be due to the fact that dietary diversity is also influenced by multiple factors. It also reinforces the recommendation that the impact of dietary diversity on the health of different populations of patients with NAFLD should be analyzed on a case-by-case basis.

To our knowledge, this study was the first to examine the relationship between salt intake, dietary diversity, and NAFLD in Chinese adults aged 18–59 years. We obtained the results using data from a large sample of Chinese medical examinations. All data were collected by professionals and quality-controlled to ensure the reliability of the information. We adjusted for multiple confounders in our analysis to build models and performed subgroup analysis to make our results more stable. In addition, our study subjects were the medical examination population, and clinical health care professionals will provide post-test health promotion education and dietary and exercise instructions to these individuals.

This study still had several limitations. First, this was a cross-sectional study, and the results did not reflect causality and the prediction of NAFLD progression, which needs to be further demonstrated in future cohort studies and interventional studies. Second, selection bias needs to be considered, and the population selected for this study was a healthy physical examination population, which may have higher health awareness than other populations. Third, this study evaluated whether the participants had NAFLD and did not assess its severity, which needs to be further explored in future studies. Fourth, the type of food intake in this study was self-reported, it might exist over-reporting and under-reporting situations, thus may causing bias to the results. Besides, the indicator of dietary diversity in this study assessed the variety of foods and did not address the daily energy intake of participants which might be a confounding factor. Finally, we used spot urine to estimate salt intake, and its assessment accuracy may be less than the gold standard.

## Conclusions

The prevalence of NAFLD was 32.48% among adults aged 18–59 years in the two Chinese physical examination centers in this study. Salt intake was associated with dietary diversity and NAFLD. Salt intake was an independent risk factor for NAFLD, while a sufficient DDS was a protective factor for NAFLD, and the results were equally stable in the subgroup analysis. It is important to change adverse lifestyle habits and pay attention to the control of salt intake and the balance of dietary diversity in the management of high-risk people to reduce the risk of NAFLD.

## Data Availability Statement

The raw data supporting the conclusions of this article will be made available by the authors, without undue reservation.

## Ethics Statement

The studies involving human participants were reviewed and approved by the Ethics Committee of the Third Xiangya Hospital of Central South University (No. 2020-S587). The patients/participants provided their written informed consent to participate in this study.

## Author Contributions

XL and YLi: conceptualization, formal analysis, data curation, and methodology. XL, YZ, CZ, LL, and YLu: software, validation, and visualization. YLi and JW: investigation. YLi, JW, and JX: resources. XL: writing—original draft preparation. XL, YD, and JX: writing—review and editing. JW, YD, and JX: supervision and project administration. YD and JX: funding acquisition. All authors have read and agreed to the published version of the manuscript.

## Funding

This work was supported by the Wisdom Accumulation and Talent Cultivation Project of the Third Xiangya Hospital of Central South University (No. YX202006) and the Special Funding for the Construction of Innovative Provinces in Hunan (No. 2020SK2091).

## Conflict of Interest

The authors declare that the research was conducted in the absence of any commercial or financial relationships that could be construed as a potential conflict of interest.

## Publisher's Note

All claims expressed in this article are solely those of the authors and do not necessarily represent those of their affiliated organizations, or those of the publisher, the editors and the reviewers. Any product that may be evaluated in this article, or claim that may be made by its manufacturer, is not guaranteed or endorsed by the publisher.
